# Changes in diet and physical activity resulting from the *Strong Hearts, Healthy Communities* randomized cardiovascular disease risk reduction multilevel intervention trial

**DOI:** 10.1186/s12966-019-0852-z

**Published:** 2019-10-25

**Authors:** Sara C. Folta, Lynn Paul, Miriam E. Nelson, David Strogatz, Meredith Graham, Galen D. Eldridge, Michael Higgins, David Wing, Rebecca A. Seguin-Fowler

**Affiliations:** 1 Friedman School of Nutrition, Tufts University, Boston, MA 02111 USA; 2 College of Education, Health and Human Development, Montana State University, Bozeman, MT 59717 USA; 3 Center for Rural Community Health, Bassett Research Institute, Cooperstown, NY 13326 USA; 4 Division of Nutritional Sciences, Cornell University, Ithaca, NY 14853 USA; 5 Texas A&M AgriLife Research, College Station, TX 77843 USA; 6 Exercise and Physical Activity Resource Center, University of California, La Jolla, San Diego, CA 92093-0811 USA; 7 Department of Nutrition and Food Science College of Agriculture and Life Sciences, Texas A&M University, College Station, Texas, TX 77843 USA

**Keywords:** Cardiovascular disease, Rural, Community, Nutrition, Diet, Exercise, Physical activity

## Abstract

**Background:**

Women living in rural areas face unique challenges in achieving a heart-healthy lifestyle that are related to multiple levels of the social-ecological framework. The purpose of this study was to evaluate changes in diet and physical activity, which are secondary outcomes of a community-based, multilevel cardiovascular disease risk reduction intervention designed for women in rural communities.

**Methods:**

*Strong Hearts, Healthy Communities* was a six-month, community-randomized trial conducted in 16 rural towns in Montana and New York, USA. Sedentary women aged 40 and older with overweight and obesity were recruited. Intervention participants (eight towns) attended twice weekly exercise and nutrition classes for 24 weeks (48 total). Individual-level components included aerobic exercise, progressive strength training, and healthy eating practices; a civic engagement component was designed to address social and built environment factors to support healthy lifestyles. The control group (eight towns) attended didactic healthy lifestyle classes monthly (six total). Dietary and physical activity data were collected at baseline and post-intervention. Dietary data were collected using automated self-administered 24-h dietary recalls, and physical activity data were collected by accelerometry and self-report. Data were analyzed using multilevel linear regression models with town as a random effect.

**Results:**

At baseline, both groups fell short of meeting many recommendations for cardiovascular health. Compared to the control group, the intervention group realized significant improvements in intake of fruit and vegetables combined (difference: 0.6 cup equivalents per day, 95% CI 0.1 to 1.1, *p* = .026) and in vegetables alone (difference: 0.3 cup equivalents per day, 95% CI 0.1 to 0.6, *p* = .016). For physical activity, there were no statistically significant between-group differences based on accelerometry. By self-report, the intervention group experienced a greater increase in walking MET minutes per week (difference: 113.5 MET-minutes per week, 95% CI 12.8 to 214.2, *p* = .027).

**Conclusions:**

Between-group differences in dietary and physical activity behaviors measured in this study were minimal. Future studies should consider how to bolster behavioral outcomes in rural settings and may also continue to explore the value of components designed to enact social and environmental change.

**Trial registration:**

clinicaltrials.gov Identifier: NCT02499731. Registered 16 July 2015.

## Background

Cardiovascular disease (CVD) is the leading cause of death for women in the USA, causing approximately 400,000 female deaths per year [1], and heart disease and stroke are among the leading causes of disability [2]. Annual age-adjusted death rates for heart disease are higher in nonmetropolitan areas compared to metropolitan areas [3]. There is a need to address cardiovascular risk, particularly among rural women, who face unique challenges to accessing healthcare and achieving healthy lifestyle behaviors [4].

There is strong epidemiological evidence for the contribution of diet and physical activity to both cardiovascular health and disease risk among women [5, 6]. Specific foods, such as fruits and vegetables [7–18], and overall dietary patterns, such as the Dietary Approaches to Stop Hypertension (DASH) [19, 20] and Mediterranean [21, 22] diets, are associated with reduced risk of CVD. However, few women are meeting recommendations for a heart-healthy diet. A study that used National Health and Nutrition Examination Survey data to assess diet quality among the U.S. population classified 42% of women as having a poor diet and less than 2% as having an ideal diet based on the American Heart Association (AHA) 2020 Strategic Impact Goals [23]. The difference between guidelines and intakes may be exacerbated in a rural setting. For example, there is some evidence that adults in rural areas consume fewer fruits and vegetables compared to non-rural counterparts [24].

There is likewise strong evidence for the role of physical activity in prevention of CVD. The AHA score for cardiovascular health includes meeting public health guidelines for physical activity as one of the components [5]. Studies demonstrate that higher amounts or intensities of aerobic activity confer a lower risk of CVD in adults [25]. There is also increasing evidence that resistance training provides additional benefits in reducing CVD risk among women [26]. Sedentary behaviors (sitting, television viewing, screen time, and computer use) have also been examined and appear to be independently associated with increased risk of CVD in adults [27]. Less than one-fifth of women (18%) are meeting current public health guidelines for aerobic and strengthening physical activity [2], and U.S. adults are spending 6 to 8 h per day engaging in sedentary activities [27]. Adults in rural areas are less likely to meet guidelines for aerobic physical activity [2], although there is some data suggesting that they spend more time in light intensity domestic physical activity compared to urban adults [28].

There are many barriers to consuming a heart-healthy diet and engaging in leisure-time physical activity in rural communities. Low population density typically means fewer supermarkets and fresh food markets. This can result in greater travel time (generally sitting in a vehicle), reduced overall food supply, and diminished quality, quantity, and intake of healthy foods, such fresh fruits and vegetables [29–36]. There is also evidence that access to recreational facilities and fitness classes and activities is limited [37–39] and sidewalks may be lacking [40]. A higher poverty rate [41] can also lead to decreased financial access and purchasing power for both healthier foods [30] and physical activity opportunities [42, 43]. Social and cultural norms and attitudes further challenge achieving a heart-healthy diet and engaging in leisure-time physical activity in rural areas [44–46]. Finally, at a personal level, in rural areas barriers to a healthier diet include knowledge gaps and negative perceptions about nutritious foods, including taste, cost, and preparation time [44, 45, 47, 48]; barriers to physical activity include childcare and caregiving duties, poor health, fear of injury, and lack of motivation [42, 43, 49, 50].

There are few community-based interventions designed for CVD prevention among women in the rural settings [45, 51–55]. Those that exist utilize behavioral theory, most commonly Social Cognitive Theory and the Transtheoretical Model. However, findings from a systematic review were that primary prevention programs for rural women had little effect on CVD risk factors, especially in the long-term [56]. In recent years, the social-ecological model has gained general endorsement for understanding and changing diet and physical activity behaviors [57–59], and offers promise as an approach that can account for the unique social and environmental barriers in the rural environment. However, only one of the previous studies utilized a social-ecological model [53].

The *Strong Hearts, Healthy Communities (SHHC)* program was designed to address key behavioral targets related to CVD prevention among rural women, including diet and physical activity. The intervention was rooted in the social-ecological model, whereby different components of the program targeted different levels of the model and were informed by Social Cognitive Theory [60, 61]. For example, at the individual level, the curriculum focused on experiential learning to support participants in developing knowledge, self-efficacy, and skill mastery related to diet and physical activity. At the interpersonal level, out-of-class materials were designed to help participants to engage friends and family in their new activities, thereby encouraging social support. In a civic engagement approach, participants worked together to complete food environment and physical activity assessments and to identify an issue to improve upon in the community (e.g. improving crosswalks, healthy at-work food policy). It was expected that the civic engagement activities would increase both social support and collective efficacy, and empower the women to become agents of change for their community, leading to improved food and/or physical activity environments. Civic engagement therefore could help promote built environment and policy changes that further reinforce individual-level change through reciprocal determinism.

In a cluster randomized, controlled trial *SHHC* led to improvements in weight and body mass index, C-reactive protein, AHA’s Life’s Simple 7 score, and 10-year risk of cardiovascular disease [62]. This paper expands on these data by examining the secondary outcomes of changes in diet and physical activity resulting from *SHHC*, an intervention designed with the rural context specifically in mind. It is important to understand behavioral outcomes in interventions conducted within a rural context given the specific challenges related to the achievement of a heart-healthy dietary pattern and physical activity in these settings.

## Methods

*SHHC* was tested in a cluster randomized, controlled trial. The study protocol has been previously published [63]. Randomization occurred at the town level: half of the towns in each state were randomized to the *SHHC* intervention program (*n* = 8), and half were randomized to a control program (n = 8). Towns were matched into pairs by population size, rural-urban community area score, and state, and then the Director of the Cornell Statistical Consulting Unit used JMP software (SAS Institute Inc., Cary, NC, USA) to randomly assign each site in the pair to either the intervention or the control. Study staff enrolled participants.

### Recruitment and eligibility

**Fig. 1:**
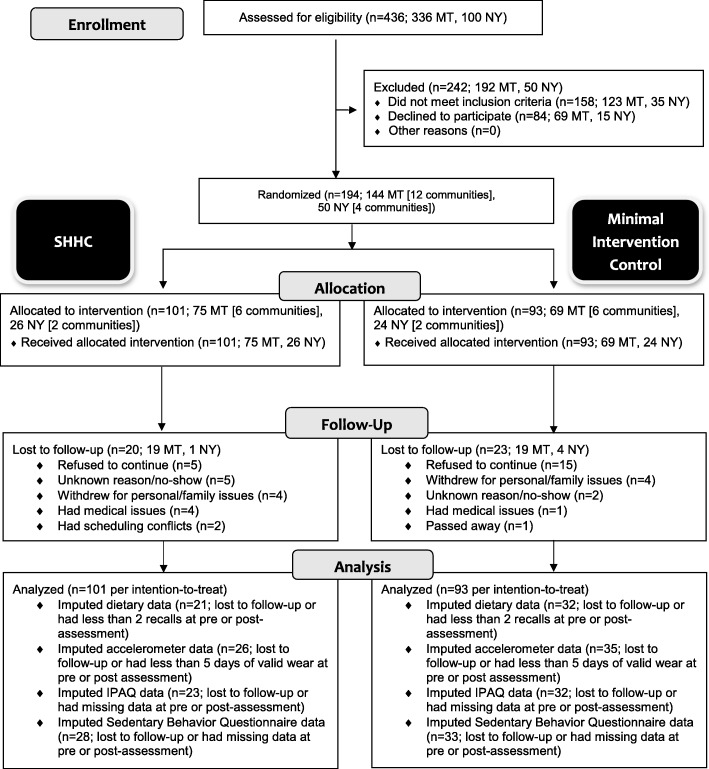
CONSORT flowchart describing progress of participants through the study. MT, Montana; NY, New York; SHHC, Strong Hearts, Healthy Communities

Towns in Montana (12 towns) and New York (4 towns) were selected by the local lead collaborators (Paul in Montana, Strogatz in New York) in partnership with the Principal Investigator (Seguin-Fowler). Towns needed to meet criteria for rurality based on Rural-Urban Commuting Area [64] and medically underserved areas or population designations [65]. Selected towns also had a county extension educator/agent (Montana) [66] or a health educator affiliated with a local healthcare system (New York) with availability, capacity, and interest in running the program. Extension educators/agents and health educators served as program leaders, rather than research personnel, with program sustainability in mind. In the planning phase of the study, local leaders were involved in community audits and focus group recruitment, and they received extensive training on the program itself. Participants were recruited through flyers, community bulletin boards, social media, radio, direct mail postcards, and newspapers, as well as through churches, healthcare providers, human services, and “word of mouth.” Inclusion criteria were female sex, age 40 years or older, overweight or obese (body mass index≥25), sedentary, English-speaking, and had physician’s approval to participate. Exclusion criteria were very high resting blood pressure (systolic pressure > 160 and diastolic pressure > 100), very low or very high resting heart rate (< 60 or > 100 beats per minute), or cognitive impairment. The selection process is depicted in Fig. 1. Reach of the *SHHC* program was calculated as the participation rate: number of enrolled *SHHC* participants in each town divided by the total number of eligible women as determined U.S. Census data on the percentage of women age 40 and over and Behavioral Risk Factor Surveillance System data on the percentage of overweight/obese adults [67]. Average reach of the *SHHC* program was 2.6% [67]. The study was approved by the Cornell University and Bassett Healthcare Network Institutional Review Boards.

### Intervention

The *SHHC* intervention curriculum was developed based on three evidence-based community programs, two of which target the individual level [51, 68] and a third, the HEART Club, which uses an innovative civic engagement approach to catalyze positive social and built environment change [69]. Civic engagement has been hypothesized to increase access to food resources even in rural food deserts [48]. *SHHC* participants met in groups by town twice per week for hourly sessions for 24 weeks (48 classes) and also attended monthly out-of-class HEART Club meetings. The intervention occurred from September/October 2015 to March–May in 2016 in Montana and November/December 2015 to June/July 2016 in New York.

The diet component aimed to change dietary patterns for alignment with DASH diet principles [70–73] and the Dietary Guidelines for Americans [74]. The nutrition behavioral aims were to increase fruits and vegetables and encourage mono- and polyunsaturated fats, lean protein, and low- and non-fat dairy; to replace refined grains with whole grains; and to decrease overall calories, desserts, processed foods, sugar-sweetened beverages, saturated fats, and sodium. The physical activities included progressive, moderate-intensity aerobic exercise (typically 20–30 min), such as walking DVDs and aerobic dance in nearly all classes; and progressive strength training (typically 10–20 min; two sets of 10 repetitions) of major muscle groups in about two-thirds of classes, utilizing exercises focusing on both single (bicep curls, chest press) and compound (squats, lunges) muscle groups. Participants were encouraged to increase the intensity of both exercise components throughout the program. Participant materials are available at http://www.strongheartshealthycommunities.org. Feasibility and sustainability in low-resource rural communities were considered by designing the program so that it could be conducted in community spaces (e.g. church basements, community meeting rooms) and by keeping equipment requirements modest (e.g. yoga mats, hand weights, DVDs).

The control program was designed to serve as a minimal-intervention attention control and was expected to improve knowledge with minimal behavioral impact. Participants in the control program met six times, once per month for an hour, during the six-month intervention period. In this program, a condensed version of *SHHC*’s curriculum information was presented using a didactic approach with no experiential activity or discussions about civic engagement. Participants did not engage in physical activity during the class sessions.

### Measures

Participants completed a questionnaire that collected basic demographic information at baseline. Demographic questions were derived from national surveys (e.g. U.S. Census). Participants were asked to complete seven dietary recalls during the four-week period just prior to the start of the intervention (“baseline”) and again in the four-week period immediately following the 24-week program (“post-intervention”). Participants were asked to wear accelerometers for seven days just prior to the start of the intervention (“baseline”) and again for seven days immediately following the conclusion of the program (“post-intervention”).

Dietary intake data were collected and analyzed using automated self-administered 24-h dietary recalls (ASA-24) [75]. Dietary data were included in analysis if at least two of the seven dietary recalls were completed at each time point to determine usual intakes of foods that are not expected to be episodic [76]. Healthy Eating Index (HEI)-2015 scores were calculated for each participant to determine alignment with the Dietary Guidelines for Americans [77]. Overall scores included 12 components: total fruits; whole fruits; total vegetables; dark green and orange vegetables and legumes; total grains; whole grains; milk; meat and beans; oils; saturated fat; sodium; and energy from solid fats, alcohol, and added sugars. These scores were then summed to derive the HEI score, which can range from 0 to 100.

Our primary measurement of physical activity was obtained using the ActiGraph Model GT3XE accelerometers (ActiGraph LLC, Pensacola, FL). Participants were instructed to wear the device at the hip for seven days and only to remove it when sleeping, bathing, or swimming. Data were recorded at 30 Hz and analyzed using an epoch length of 60 s. Data were screened using current best practices [78], and non-wear time was identified (and excluded) using a widely-used algorithm developed by Choi et al. [79]. Daily level data were excluded if wear time was less than 10 h (600 min) in a day, and participant level data were only included if the participant had five or more valid days of wear (i.e. ≥3000 min across five days with ≥600 min each), or four valid days of wear with at least 750 min per day. Because participants were essentially healthy and without disability, Freedson cut-points were used to determine minute-level intensity of physical activity [80]. Step counts were also determined. A categorical variable was created from the accelerometer data for both pre- and post-intervention: average moderate or vigorous physical activity (MVPA) minutes per day as measured by accelerometer was multiplied by 7 to give average minutes of MVPA per week. If average minutes of MVPA per week was greater than or equal to 150 min then the participant is meeting the physical activity recommendations [25]. If average minutes of MVPA is less than 150 min per week then the participant is not meeting physical activity recommendations.

We used self-report as a secondary measure of physical activity to help account for the limitations of accelerometry, such as an inability to capture some types of activities (those involving use of the upper extremities, stationary activities, and swimming), and an inability to distinguish the purpose of the physical activity (work, leisure, transportation) [81]. Self-report measures complement objective measures by accounting for these limitations, although they have their own limitations such as recall bias and an inability to account for shorter durations or lower-intensity activities. The International Physical Activity Questionnaire Short Form (IPAQ-SF) was used pre- and post-intervention to collect self-report of physical activity [82–84]. Metabolic equivalent (MET) minutes per week were compiled according to the IPAQ’s Guidelines for Data Processing and Analysis [85]. Self-report of sedentary time was obtained using the Sedentary Behavior Questionnaire [86].

### Statistical analysis

Descriptive statistics for the whole sample and by treatment groups were compiled and tabulated. Comparisons of continuous and categorical variables between the groups at baseline were done using t-tests and chi-square tests, respectively. Since the observations are clustered by town, we conducted multilevel linear regression models where town was treated as a random effect. For each diet and physical activity outcome, an unadjusted model was run with pre-post intervention change as the dependent variable, with treatment as a fixed effect and site as a random effect. Adjusted models, which additionally controlled for baseline values of the outcome, age, marital status, and education, were estimated. Missing data were handled using multiple imputation to minimize bias which could have resulted if complete case analysis were used. The imputation was conducted in SAS (PROC MI). Thirty datasets were imputed and SAS PROC MIANALYZE was then used to combine the model results from within each imputed data set into one summary output. We also used multilevel logistic regression to assess whether treatment was associated with meeting the MVPA recommendation (as measured by accelerometry). Generalized linear mixed effects model (PROC GLIMMIX in SAS) was used with site as a random effect, where meeting the recommendation at outcome was a 1 and not meeting a recommendation at outcome was a 0; education, age, marital status, and baseline meeting of recommendation were included as covariates. All tests were two-sided. We adjusted for multiple testing using the Benjamini-Hochberg approach [87] to avoid risk of an inflated type I error based on the large number of significance tests. We applied the method to physical activity and dietary outcomes together and used a false positive rate of 20%. The adjusted *p*-value for significance based on correcting for both the number of within arm and between arm tests (37 outcomes with three statistical tests each for a total of 111 tests) is *p* = .0468. Analyses were conducted using SAS version 9.4 (SAS Institute Inc., Cary, NC, USA).

## Results

Of a total of 194 study participants, 141 had pre-post 24-h dietary recall data and 133 had pre-post accelerometry data; missing data were imputed (Fig. 1). There were no statistically significant differences in demographic characteristics between the intervention and control group in the analytic sample (Table 1). At baseline, 28% of participants (*n* = 54) completed seven dietary recalls and 71% (*n* = 137) completed at least five dietary recalls; 46% (*n* = 90) had a full seven days of valid accelerometer wear. There was a statistically significant difference in age at baseline between participants who had completed at least two dietary recalls at post-intervention and those who had not (59.6 years for completers vs. 56.5 for non-completers, *p* = .03). There were no statistically significant differences between those who had complete pre-post accelerometry data and those who did not. There were no statistically significant differences in baseline dietary (Table 2) or physical activity (Table 3) measures between the intervention and control groups (*p ≥* .05 in all cases).

**Table 1:** Baseline Characteristics of Participants by Intervention Condition

Characteristic	Total (*n* = 173 in 16 towns)	Control (*n* = 81 in 8 towns)	SHHC (*n* = 92 in 8 towns)	p-value
Age, mean (SD)	58.6 (9.5)	59.0 (96)	58.9 (9.5)	.98
Income, n (%)^a^				.24
< $25,000	33 (21)	12 (16)	21 (26)	
$25,000–$50,000	47 (30)	26 (34)	21 (26)	
> $50,000	78 (49)	39 (50)	39 (48)	
Marital status, n (%)^b^				.61
In a relationship (married or member of an unmarried couple)	122 (72)	57 (70)	65 (74)	
Not in a relationship (divorced, widowed, separated, or never been married)	47 (28)	24 (30)	23 (26)	
Educational level, n (%)^c^				.88
High school or less	38 (23)	17 (21)	21 (24)	
Technical or vocational school/some college	52 (31)	24 (30)	28 (32)	
College graduate	52 (31)	26 (32)	26 (30)	
Postgrad/professional	26 (15)	14 (17)	12 (14)	
Racial/ethnic minority, n (%)^c^	9 (5)	4 (5)	5 (6)	.84
Employment status, n (%)^d^				.10
Employed for wages or self-employed	120 (71)	62 (77)	58 (65)	
Not working or retired	50 (29)	19 (23)	31 (35)	
Smoking^c^	7 (13)	3 (12)	4 (14)	.81
Body mass index, mean (SD)	35.1 (6.3)	35.4 (6.7)	34.8 (6.0)	.56
Weight, mean (SD), kg	93.7 (17.7)	95.6 (19.0)	92.0 (16.1)	.19
% meeting guidelines for physical activity^e^	19.7	19.3	20.0	.91

^a^Total *n* = 158 (15 missing)

^b^Total *n* = 169 (4 missing)

^c^Total *n* = 168 (5 missing)

^d^Total *n* = 170 (3 missing)

^e^Total *n* = 162 (11 missing)

**Table 2:** Dietary Outcomes at Baseline and Post-Intervention by Treatment Group

Outcome	Baseline Mean (SD)		Post-intervention, Mean (SD)		Pre-post change					
					Control		*SHHC* Intervention		Adjusted difference^b,c^	
	Control	*SHHC* Intervention	Control	*SHHC* Intervention	Mean (95% CI) ^a^	p-value	Mean (95% CI) ^a^	p-value	Mean (95% CI)	p-value
Kcal	1826.4 (59.2)	1744.2 (48.9)	1560.3 (65.6)	1562.8 (54.0)	***−233.5 (− 389.0,-78.0)***	***.003***	***−183.9 (− 332.3,-35.4)***	***.015***	49.6 (− 144.3, 243.6)	.616
Carbohydrate, g	198.7 (6.9)	190.3 (6.3)	168.4 (7.9)	174.7 (7.3)	***−26.8 (−46.9,-6.8)***	***.009***	−16.4 (−36.5,3.8)	.111	10.4 (− 15.6,36.5)	.432
Protein, g	75.8 (2.2)	73.2 (1.8)	72.9 (2.8)	70.0 (2.3)	−1.5 (−7.1,4.1)	.593	−2.7 (−7.6,2.3)	.289	−1.2 (− 7.7,5.4)	.729
Fiber, g	15.6 (0.7)	15.2 (0.5)	14.5 (0.8)	15.9 (0.8)	−0.9 (−2.9,1.1)	.355	0.7 (−1.2,2.6)	.461	1.6 (−0.8,4.1)	.195
Total fat, g	79.5 (3.2)	76.1 (2.5)	66.1 (3.6)	65.8 (2.8)	***−12.1 (−20.6,-3.6)***	***.005***	***−10.5 (− 18.3,-2.6)***	***.009***	1.7 (−8.9,12.2)	.757
Saturated fat, g	26.9 (1.2)	26.2 (1.0)	22.9 (1.3)	21.9 (1.1)	***−3.6 (−6.8,-0.5)***	***.023***	***−4.4 (−7.2,-1.6)***	***.004***	1.4 (−2.4,5.1)	.731
Monounsaturated fat, g	28.7 (1.1)	27.4 (0.9)	22.5 (1.2)	23.2 (1.0)	***−5.8 (−8.8,-2.7)***	***<.001***	***−3.2 (− 5.8,-0.6)***	.***002***	1.9 (−1.6,5.5)	.479
Polyunsaturated fat, g	17.1 (0.9)	16.1 (0.6)	15.2 (1.0)	15.4 (0.7)	−1.5 (−3.8,0.9)	.233	−0.4 (−2.5,1.6)	.684	1.0 (−1.8,3.9)	.481
Dietary cholesterol, mg	274.0 (12.4)	281.6 (14.6)	263.5 (19.7)	267.0 (14.1)	−10.4 (− 54.8,33.9)	.644	−9.7 (− 50.4,31.0)	.640	0.7 (− 55.0,56.5)	.979
Sodium, mg	3146.7 (104.0)	3118.3 (89.2)	2841.6 (116.3)	2775.5 (91.5)	***− 266.4 (− 505.7,-27.0)***	***.029***	***− 299.6 (− 525.1,-74.1)***	***.009***	−33.2 (− 331.0,264.6)	.827
HEI 2015 Score	56.3 (1.3)	57.0 (1.1)	57.6 (1.6)	61.9 (1.4)	1.0 (−2.8,4.8)	.603	***4.9 (1.4,8.4)***	***.006***	3.9 (−1.0,8.8)	.119
Average daily intake of…										
Fruits and vegetables in cup equivalents	2.6 (0.1)	2.6 (0.1)	2.2 (0.2)	2.8 (0.2)	***−0.5 (−0.8,−0.1)***	***.023***	0.1 (− 0.2,0.5)	.529	**0.6 (0.1,1.1)**	**.026**
Fruits in cup equivalents	0.9 (0.1)	1.0 (0.1)	0.8 (0.1)	1.1 (0.1)	-0.1 (−0.4,0.2)	.348	0.1 (−0.2,0.4)	.401	0.3 (−0.1,0.6)	.179
Vegetables in cup equivalents	1.7 (0.1)	1.6 (0.1)	1.4 (0.1)	1.7 (0.1)	***−0.3 (−0.5,− 0.1)***	***.004***	0.0 (− 0.2,0.2)	.866	**0.3 (0.1,0.6)**	**.016**
Whole grains in oz. equivalents	0.8 (0.1)	0.8 (0.1)	1.0 (0.1)	0.9 (0.1)	0.2 (−0.1,0.5)	.230	0.1 (−0.1,0.4)	.360	-0.1 (−0.4,0.3)	.738
Refined grains in oz. equivalents	4.4 (0.3)	3.9 (0.2)	3.6 (0.3)	3.3 (0.2)	−0.6 (−1.2,0.1)	.109	−0.6 (−1.3,0.0)	.058	− 0.1 (− 0.9,0.8)	.871
Seafood high in n-3 fatty acids in oz. equivalents	0.0 (0.0)	0.1 (0.0)	0.2 (0.1)	0.2 (0.1)	0.1 (0.0,0.3)	.170	0.1 (0.0,0.2)	.069	0.0 (−0.2,0.2)	.736
Legumes in oz. equivalents	0.1 (0.0)	0.1 (0.0)	0.1 (0.0)	0.1 (0.0)	0.0 (0.0,0.0)	.926	0.0 (0.0,0.1)	.237	0.0 (0.0,0.1)	.335
Oils in grams^d^	22.8 (1.4)	19.8 (1.0)	19.9 (1.5)	19.9 (1.3)	−1.7 (−5.2,1.8)	.333	0.0 (−3.5,3.4)	.981	1.7 (− 3.0,6.4)	.481
Solid fats in grams^e^	38.9 (1.8)	38.9 (1.7)	31.5 (2.0)	29.7 (1.8)	***−7.6 (−12.7,-2.5)***	***.004***	***−9.2 (−14.4,-4.1)***	***<.001***	−1.7 (−8.6,5.2)	.632
Added sugars in teaspoon equivalents	11.9 (0.9)	11.9 (0.8)	9.9 (1.0)	10.1 (0.9)	−2.2 (−4.8,-0.3)	.087	−1.9 (−4.3,0.5)	.123	0.3 (−3.0,3.7)	.847

^a^Significant within-group pre-post changes are bolded and italicized (corrected *p*-value using Benjamini-Hochberg approach is .0468)

^b^Significant between-group differences are bolded (corrected *p*-value using Benjamini-Hochberg approach is .0468)

^c^Adjusted for town, education, age, marital status, and baseline value of the outcome

^d^Fats naturally present in nuts, seeds, seafood; unhydrogenated vegetable oils, except palm oil, palm kernel oil, coconut oils; fat in avocado and olives above allowable amount; 50% of fat present in stick/tub margarines, margarine spreads (grams)

^e^Fats naturally present in meat, poultry, eggs, dairy (lard, tallow, butter); hydrogenated/partially hydrogenated oils; shortening, palm, palm kernel, coconut oils; coconut meat, cocoa butter; 50% of fat in stick/tub margarines, margarine spreads (grams)

**Table 3:** Physical Activity Outcomes at Baseline and Post-Intervention by Treatment Group

Outcome	Baseline Mean (SE)		Post-intervention Mean (SE)		Pre-post change					
					Control		*SHHC* Intervention		Adjusted difference^b,c^	
	Control	*SHHC* Intervention	Control	*SHH* Intervention	Mean (95% CI) ^a^	*p-value*	Mean (95% CI) ^a^	p-value	Mean (95% CI)	p-value
Accelerometer										
Average daily step count	4687.9 (243.2)	4698.1 (718.2)	5220.5 (325.2)	5870.5 (304.5)	330.5 (− 626.5, 1287.4)	.498	912.4 (−498.1, 2322.8)	.201	581.9 (− 976.8, 2140.6)	.462
Light activity, average min/day	305.4 (9.3)	307.0 (8.8)	313.3 (11.6)	328.1 (9.9)	4.9 (−27.0, 36.8)	.764	16.1 (−13.9, 46.0)	.292	11.2 (−29.3, 51.6)	.588
MVPA, average min/day	14.2 (1.4)	14.6 (1.5)	17.0 (2.5)	22.1 (2.6)	2.5 (−4.2, 9.1)	.468	***7.3 (0.9, 13.7)***	***.026***	4.8 (−3.5, 13.2)	.254
Sedentary time, average min/day	521.5 (10.2)	503.3 (9.3)	504.7 (13.0)	500.3 (11.5)	−13.9 (−41.9, 14.0)	.328	−6.0 (−34.6, 22.5)	.679	7.9 (−31.2, 47.0)	.690
% of activity that is light intensity	36.2 (1.0)	37.0 (0.9)	37.4 (1.2)	38.6 (1.0)	0.9 (−2.2, 3.9)	.569	1.3 (−1.7, 4.3)	.388	0.4 (−3.6, 4.4)	.831
% of activity that is MVPA	1.7 (0.2)	1.8 (0.2)	2.0 (0.3)	2.6 (0.3)	0.3 (−0.4, 1.0)	.370	***0.8 (0.1, 1.5)***	***.025***	0.5 (−0.4, 1.4)	.307
% of total activity time that is sedentary	62.1 (1.0)	61.2 (0.9)	60.6 (1.3)	58.9 (1.2)	−1.2 (−4.3, 1.9)	.454	−2.1 (−5.2, 1.0)	.182	−0.9 (−5.0, 3.2)	.655
IPAQ										
Total MET- min/week	593.8 (98.1)	702.0 (117.0)	1181.0 (281.0)	2080.4 (304.0)	499.2 (−150.5, 1148.8)	.132	***1296.6 (662.7, 1930.5)***	***<.0001***	797.4 (−92.4, 1687.2)	.079
Walking MET- min/week	93.0 (18.0)	135.0 (23.9)	213.8 (31.4)	346.1 (31.0)	***108.2 (30.9, 185.5)***	***.006***	***221.7 (141.3, 302.2)***	***<.0001***	**113.5 (12.8, 214.2)**	**.027**
Moderate MET-min/week	197.6 (41.6)	277.4 (65.8)	432.0 (129.2)	548.1 (100.8)	168.1 (−109.4, 445.6)	.234	***246.2 (8.6, 483.9)***	***.042***	78.2 (− 260.9, 417.3)	.651
Vigorous MET- min/week	303.2 (73.8)	289.5 (71.4)	535.1 (187.8)	1186.2 (243.4)	182.7 (− 298.2, 663.6)	.456	***836.1 (329.1, 1343.1)***	***.001***	653.4 (−38.8, 1345.6)	.064
Sedentary Behavior Questionnaire										
Total hours per week of sedentary time	75.6 (6.5)	76.7 (4.8)	81.5 (8.6)	79.9 (16.7)	2.4 (−24.1, 28.9)	.857	0.0 (−35.2, 35.2***)***	.998	−2.5 (−43.5, 38.6)	.905
Hours per week sitting at desk	25.5 (2.3)	21.2 (1.8)	29.2 (3.6)	19.7 (2.4)	4.4 (−3.4, 12.1)	.270	−3.7 (−10.5, − 3.1)	.288	−8.0 (− 17.7, − 1.6)	.101
Hours per week sitting with friends	10.6 (1.7)	10.2 (1.2)	11.2 (3.4)	13.0 (6.3)	0.3 (−9.9, 10.4)	.957	1.8 (− 8.6, 12.3)	.729	1.6 (13.3, 16.5)	.836
Hours per week sitting and reading	26.6 (2.6)	26.8 (2.4)	25.3 (2.8)	23.7 (2.7)	−1.6 (−7.6, 4.3)	.593	−3.5 (−10.0, 3.1)	.296	−1.8 (− 10.4, 6.7)	.670
Hours per week driving	13.0 (3.7)	18.5 (2.4)	15.7 (4.9)	23.6 (13.9)	−0.1 (−20.6, 20.3)	.990	5.5 (− 24.5, 35.6)	.714	5.7 (− 25.5, 36.8)	.720

^a^Significant within-group pre-post changes are bolded and italicized (corrected p-value using Benjamini-Hochberg approach is .0468)

^b^Significant between-group differences are bolded (corrected p-value using Benjamini-Hochberg approach is .0468)

^c^Adjusted for town, education, age, marital status, and baseline value of the outcome

For both groups, on average, intakes of sodium, added sugars, fiber, and fruits and vegetables failed to meet recommendations for cardiovascular health [88, 89] at baseline (Table 2). The average HEI diet quality score placed participants in both groups slightly below the U.S. national average of 59 [90].

Both groups also fell short of the public health recommendation for physical activity at baseline. While 150 min per week of at least moderate activity or 75 min per week of vigorous activity is recommended [91], participants obtained closer to 100 min per week of MVPA, with about half as moderate activity, based on daily averages (Table 3). Approximately 20% of participants were meeting the recommendation and there were no differences between arms at baseline (Table 1). Average daily step counts were approximately half of the widely-promoted recommendation of 10,000 steps per day [92].

Compared to the control group, the intervention group realized statistically significant improvements in intake of fruit and vegetables combined (difference: 0.6 cup equivalents per day, 95% CI 0.1 to 1.1, *p* = .026) and in vegetables alone (difference: 0.3 cup equivalents per day, 95% CI 0.1 to 0.6, *p* = .016) (Table 2). For physical activity, there were no statistically significant differences between the intervention and control groups based on accelerometry, our primary measure of physical activity (Table 3). By self-report, compared to the control group, the intervention group experienced a greater increase in walking MET-minutes per week (difference: 113.5 MET-minutes per week, 95% CI 12.8 to 214.2, *p* = .027). No other statistically significant differences in diet or physical activity outcomes between groups were observed.

## Discussion

This study helps elucidate behavioral outcomes of *Strong Hearts, Healthy Communities*, one of the first multi-level community-based CVD prevention interventions for rural women. Baseline data from this study confirm the need for interventions to improve behaviors to reduce risk of cardiovascular disease in this population of rural women. Intakes of salt and added sugars were both approximately double AHA recommendations, and participants consumed less fruit, vegetables, and fiber than recommendations. Average minutes of at least moderate physical activity per week were below the recommended 150; at baseline only approximately one-fifth of study participants were meeting these recommendations as measured by accelerometer.

Results suggest minimal between-group behavioral changes in this study. For dietary outcomes, the between-group changes were statistically significant for both fruits and vegetables combined and vegetables alone. These changes reflect a statistically significant within-group decrease in the control group rather than an increase in the *SHHC* group. For physical activity outcomes, no between-group differences by accelerometry were statistically significant, however there was a statistically significant between-group change in walking MET-minutes per week by self-report. In the primary trial report, there were statistically significant between-group differences in weight and body mass index and improvement in C-reactive protein [62]. The behavioral data do not correspond well to those findings.

A possible explanation for the discrepancy is that between-group comparisons were diluted by improvements in the control group for several dietary outcomes, including total calories, as revealed by statistically significant within-group results. The minimal-intervention attention control program was designed to provide basic information across a total of six contact hours and implemented based upon community partner preferences and feasibility (versus no program or a delayed program delivery for controls). Both program curricula provided information about CVD rates among women, risk factors, and the basics of a heart-healthy lifestyle including healthy eating and physical activity information. Core educational elements that were common across both programs, particularly around diet, show promise for effectively changing behavior.

For physical activity outcomes, although non-significant, between-group differences favored *SHHC*. It is possible that there was insufficient power to detect changes in these secondary outcomes and therefore an increased probability of a Type II error. For example, per our data for average MVPA, there was an effect size of 0.73 with a standard deviation of 6.4. We therefore would have needed a sample size of 510 individuals to achieve 80% power at a *p*-value of .05 and accounting for clustering with an intra-class correlation coefficient of .025. The possibility of a Type II error accounting for the discrepancy between the primary trial report findings and the behavioral outcomes presented here is further supported by findings from this trial indicating that the between-group change in weight was largely accounted for by a change in aerobic fitness, as measured by a step test [93].

Although not our primary measure of physical activity, there were between-group differences in walking MET-minutes per week by self-report. While both the *SHHC* and control curricula provided general information about the benefits of physical activity, the *SHHC* program included the use of walking DVDs in class and emphasized alternative options for out-of-class walking, such as community recreational centers. It is also possible that engagement in HEART Club activities contributed to increased walking. In one study, volunteering, a form of civic engagement, led to increased walking among older women [94].

It is important to consider whether issues with implementation of *SHHC* could help explain the minimal between-group behavioral results. Although sites were randomized, there could be other factors that influence results that were not accounted for due to the number of sites. However, process evaluation data do not support this possibility. Program leaders had high levels of adherence to the *SHHC* curriculum (fidelity greater than 80%), with exception of only one of the eight intervention sites (68.9%) [67]. The dose delivered and class effectiveness ratings were also high at all sites except one [67]. Strong implementation of the control curriculum (average 90% with high levels across all sites, unpublished data) likely contributed to the favorable within-group dietary changes observed in the control group that may help explain the lack of between-group changes in many of these outcomes.

This study adds to the small body of evidence on behavioral interventions designed specifically for rural women. *Heart Smart for Women* [54] included behavioral strategies that were similar to *SHHC*; however it was not designed as a multilevel intervention. In that study, there were modest pre-post changes in several outcomes, including fruit consumption and moderate intensity physical activity. A perceived lack of resources, including sources of healthy food, gyms, and safe walking paths, was reported in focus groups conducted with women from the counties where *Heart Smart for Women* was conducted. In a study conducted in rural counties in upstate New York and Virginia that, like *SHHC,* used a social-ecological model, participating women attended a single community-visioning meeting that resulted in a request for community-level changes that were then implemented by a community organization [53]. Community-level changes were modest, however. For example, in New York, physical activity resources were listed on a website. Favorable pre-post changes in fruit and vegetable intake were realized, although these were greater in a group that also included visits by registered nurses to focus on individual-level changes. In this study, HEART Club successes included organizing county-wide health fairs and a restaurant healthy food labeling initiative [62].

Taken together, our results and those of prior studies suggest that while it is possible to achieve good implementation of interventions in the rural environment, attainment of robust behavioral outcomes remains a challenge. The core educational elements from *SHHC* related to diet and cardiovascular disease provide a basis on which future interventions can build. A multilevel approach was acceptable to participants and they achieved some change in their communities, suggesting that this remains a promising approach. Future studies could continue to explore the value of components designed to enact social and environmental change to better contribute to individual-level behavior change.

This study has several strengths and limitations. It was conducted in multiple rural underserved communities across two states in different regions of the U.S. Thus, findings may generalize to other rural settings. While the study population was predominantly white, it reflected the racial/ethnic composition of the rural communities in which the research was conducted. However because of this it is possible the results will not generalize to other populations. The 24-h dietary recall methodology, IPAQ-SF, and Sedentary Behavior Questionnaire have all been validated [84, 86, 95]. Nevertheless, findings are limited by the self-reported nature of these data. Participants were aware of the timeframe during which they would complete the questionnaires and may have made changes in diet and physical activity based on this (reactivity). There were seasonal differences in the pre and post timeframes that likely affected outcomes. For the majority of participants, the baseline period was in September/October, when produce is being harvested and the weather is conducive to outdoor activities, including walking; and post measurements were conducted in March through May, when fresh produce is much less available and the weather is less favorable for outdoor physical activity. This timing may be responsible for the decreases in fruit and vegetable intakes noted in the control group, and may suggest success of the *SHHC* curriculum in providing women with the behavioral strategies needed to maintain fruit and vegetable intakes despite the seasonal lack of availability.

## Conclusions

Heart disease is an important issue to address in rural communities. There is a need for interventions that address the many barriers to achieving heart-healthy behaviors, particularly because access to healthcare can be extremely limited. The *SHHC* curriculum achieved success in changing health outcomes [62], however these changes remain largely unexplained in terms of the antecedent diet and physical activity behaviors. Future studies should consider how to bolster behavioral outcomes, possibly by including more and different strategies for affecting multilevel change.

## Data Availability

The datasets used and analyzed in the current study are available on reasonable request.

## Abbreviations

AHA: American Heart Association
ASA-24: automated self-administered 24-h dietary recall
CI: confidence interval
CVD: cardiovascular disease
DASH: Dietary Approaches to Stop Hypertension
HEI: Healthy Eating Index
IPAQ-SF: International Physical Activity Questionnaire Short Form
MET: metabolic equivalents
MVPA: moderate or vigorous physical activity
SHHC: Strong Hearts, Healthy Communities
USA: United States of America
